# Growth of Murine Splenic Tissue Is Suppressed by Lymphotoxin β-Receptor Signaling (LTβR) Originating from Splenic and Non-Splenic Tissues

**DOI:** 10.1371/journal.pone.0166901

**Published:** 2016-12-09

**Authors:** Novica M. Milićević, Klaus Nohroudi, Friederike Schmidt, Hendrik Schmidt, Cornelia Ringer, Grith Lykke Sorensen, Živana Milićević, Jürgen Westermann

**Affiliations:** 1 Institute of Histology and Embryology, Faculty of Medicine, University of Beograd, Beograd, Serbia; 2 Department I of Anatomy, University of Cologne, Cologne, Germany; 3 Center for Structural and Cell Biology in Medicine, Institute of Anatomy, University Lübeck, Lübeck, Germany; 4 Department of Cancer and Inflammation, Institute of Molecular Medicine, University of Southern Denmark, Odense, Denmark; Tokyo Daigaku, JAPAN

## Abstract

Development and maintenance of secondary lymphoid organs such as lymph nodes and spleen essentially depend on lymphotoxin β-receptor (LTβR) signaling. It is unclear, however, by which molecular mechanism their size is limited. Here, we investigate whether the LTβR pathway is also growth suppressing. By using splenic tissue transplantation it is possible to analyze a potential contribution of LTβR signaling inside and outside of the implanted tissue. We show that LTβR signaling within the endogenous spleen and within non-splenic tissues both significantly suppressed the regeneration of implanted splenic tissue. The suppressive activity positively correlated with the total number of LTβR expressing cells in the animal (regenerate weights of 115 ± 8 mg in LTβR deficient recipients and of 12 ± 9 mg in wild-type recipients), affected also developed splenic tissue, and was induced but not executed via LTβR signaling. Two-dimensional differential gel electrophoresis and subsequent mass spectrometry of stromal splenic tissue was applied to screen for potential factors mediating the LTβR dependent suppressive activity. Thus, LTβR dependent growth suppression is involved in regulating the size of secondary lymphoid organs, and might be therapeutically used to eradicate tertiary lymphoid tissues during autoimmune diseases.

## Introduction

Secondary lymphoid organs such as lymph nodes and spleen are essential sentinels against invading pathogens and developing tumors [1, 2]. They facilitate activation and clonal expansion of T cells, as well as their subsequent interaction with B cells [3, 4] which is necessary for pathogen recognition [5], elimination, and memory formation [6]. The lymphotoxin β-receptor (LTβR) pathway is not only involved in the export of T cells from the thymus [7], but is one of the main regulators of secondary lymphoid organ formation [8, 9, 10, 11]. It is expressed in lymphoid but also non-lymphoid organs [12] by non-hematopoietic stromal cells [13], endothelial cells [14], dendritic cells [15], and monocytes [16, 17]. LTβR has two membrane bound ligands LTα_1_β_2_ and LIGHT which are preferentially expressed in lymphoid organs [18] by hematopoietic cells such as lymphoid tissue inducer cells [8, 19], dendritic cells [20], and activated T and B cells [21]. After ligand binding secretion of chemokines (e.g. CCL19, CCL21, CXCL13) [22] and expression of adhesion molecules (e.g. ICAM-1, VCAM-1, MAdCAM-1) are up-regulated thereby attracting more ligand bearing cells [23]. This establishes a positive feed-back loop that is essential for the development and maintenance of secondary lymphoid organs [8, 24]. The importance of this molecular pathway for secondary lymphoid organ development is demonstrated by LTβR deficient mice that lack lymph nodes and Peyer’s patches [25, 26]. In addition, splenic white pulp structure is grossly altered [27]: T- and B-cell zones are not separated from each other and no marginal zone is present [28, 25, 8]. This leads to a severe impairment of the immune response [28, 25, 8].

An unresolved issue is by which molecular mechanism the growth of secondary lymphoid organs is terminated. Some kind of control must be present, since during development the individual secondary lymphoid organs increase in size, but at a certain point their growth is halted. Lymphocytes seem to be able to sense their density [29] and stop proliferation at a threshold level (quorum sensing). For example, the more IL-2 is produced by CD4 T cells, the more active are regulatory T cells that suppress further CD4-T-cell expansion [30]. In addition, B-cell derived antibodies are able to suppress B-cells proliferation [31].

In the present study we asked whether the non-lymphocyte compartment is also involved in controlling organ size. Given the importance of the LTβR pathway for secondary lymphoid organ development, we investigated whether the LTβR pathway, apart from its essential growth-supporting function, is also involved in suppressing the growth of secondary lymphoid tissues. Since LTβR deficient mice completely lack lymph nodes and Peyer’s patches, the spleen was studied. In order to be able to differentiate between supporting and suppressive contributions of the LTβR pathway, we used the well-established approach of splenic tissue transplantation [32, 33]. By using wild-type and LTβR deficient mice as donors and recipients of splenic tissue and performing cross-transplantations, it is possible to analyze a potential contribution of LTβR expression inside and outside the transplanted splenic tissue.

Our data confirm that LTβR signaling within splenic tissue is essential for its regeneration. In addition, we show for the first time that LTβR signaling also suppresses the regeneration of implanted and reduces the size of developed splenic tissue, with the extent of growth suppression positively correlating with the number of LTβR expressing cells present in the animal.

## Materials and Methods

### Animals

C57BL/6 wild-type, LTβR deficient [25], and MFAP4 deficient [34] mice were used. Wild-type and LTβR deficient mice were obtained from Charles River GmbH (Sulzfeld, Germany) and MFAP4 deficient mice from Odense [34]. Mice of both sexes (8 to 12 weeks old) were used in the experiments (in total n = 179). The animals were housed and bred under specific pathogen-free conditions in the Central Animal Facility of the University of Lübeck (Germany) and sacrificed by cervical dislocation. Permission for the animal experiments was issued by the animal care and use committee (Kiel, Germany, V252-72241.122-1/24-3/02).

### Transplantation of splenic tissue

Surgery was performed under clean but not sterile conditions [33]. Mice were anaesthetized with ketamine hydrochloride (12 mg/ml) and xylazine hydrochloride (1.6 mg/ml) dissolved in 0.9% NaCl with a dosage of 10 ml/kg body weight. The abdomen of the recipient was opened and the endogenous spleen was removed (splenectomy) by cutting the gastro-splenic ligament and the splenic blood vessels as described [32]. Then the donor spleen was obtained, cut in 4 equal pieces, and two of them (corresponding to 50% of the normal spleen) were implanted into the greater omentum of the splenectomized recipients. The abdomen was closed by suturing muscle and skin layers separately with a continuous absorbable suture (5–0, ethicon vicryl) finally covering the surface by plaster spray. Eight weeks later the splenic regenerates were removed.

### Antibodies

The following cell populations were identified by immunohistochemistry: T cells (TCRβ^+^) and B cells (B220^+^). With flow cytometry the following molecules were identified: CD4 (RM4-5), CD25 (7D4), B220 (RA3-6B2), IgD (11-26c.2a), TCRβ (H57-597), isotype control (R35-95).

### Histology

Splenic tissue was embedded in Tissue-Tek^®^ at -20°C and frozen sections (12 μm) were prepared. Immunohistochemistry was performed as described [33, 35]. In brief, cryostat sections were air-dried at room temperature for 2 hours, and fixed in methanol-acetone (1:1 (v/v), 10 minutes at –20°C) followed by fixation in 4% paraformaldehyde (45 minutes at 4°C). Sections were incubated with the appropriate dilution of primary antibody for 1 hour followed by secondary reagents with an incubation time of 30 minutes each. Labelled cells were revealed either by the Fast Blue or diaminobenzidine reaction. The sections were mounted with water-soluble medium and cover-slipped.

The area of the different compartments was determined using an Axiovert 200/HAL 100 microscope (Zeiss, Jena, Germany) and Palm@Robo V2.2.2 software (P.A.L.M Microlaser Technologies AG, Bernried, Germany) and expressed as percentage of the total surface area. The area of the mixed T/B-zone was determined as follows. First, splenic sections were stained for either T cells or B cells and the positively stained areas were determined (value 1 and value 2). On a consecutive section T and B cell staining was performed together and the resulting area was measured (value 3). By adding value 1 and 2, and then subtracting value 3 the size of the mixed T/B-zone was obtained. These results were confirmed by simultaneously labelling T and B cells with different fluorescent dyes (green and red, respectively) and analyzing the overlapping area by confocal laser microscopy [36].

### Flow cytometry

Splenic tissues were weighed and single cell suspensions prepared. After removal of red blood cells leukocyte numbers were determined and the cells were stained [37]. Flow cytometry was performed on a FACS Calibur™ (BD Biosciences), and acquisition and analysis of the data was done using CellQuest™Pro (Version 4.0.2, BD Biosciences, Heidelberg, Germany).

### Quantitative real-time RT-PCR

RNA was isolated with the innuPREP RNA mini kit (Analytik Jena AG, Jena, Germany). The cDNA-Synthese Kit (#EP 0452, Thermo Scientific, Waltham, USA) was used with an additional DNAse step (DNase I, 1U/μl, Sigma-Aldrich, Inc.) for the preparation of nucleotides for quantitative real-time RT-PCR. The PCR was conducted using SYBR Green or TaqMan PCR Master Mix (Life Technologies, Darmstadt, Germany) on the SDS ABI 7900 system (Applied Biosystems, Darmstadt, Germany). For RORγt detection, TaqMan gene expression single-tube assay (Invitrogen, Darmstadt, Germany) was used (*probe*: 5´CCT CTA CCC CGA CAT TCC CAA GGA GGA GGC). All other primers were designed using CloneManager 7.01 (SciEd, Cary, NC). Primer sequences were: CCL19 *forward*: 5´-TGA TGC GGA AGA CTG CTG and *reverse*: 5´-CTT TCA CGA TGT TCC CAG GG; CCL21 *forward*: 5´-AGG CAG TGA TGG AGG GGG T and *reverse*: 5´-CTT TCA CGA TGT TCC CAG GG; CXCR5 *forward*: 5´-CCT GCT GCT GGC CTG TAT AG and *reverse*: 5´-CCA ACC TTG GCA AAG AGG AG; CXCL13 *forward*: 5’-CAT AGA TCG GAT TCA AGT TAC GCC and *reverse*: 5’-TCT TGG TCC AGA TCA CAA CTT CA; IL-7 *forward*: 5´-GGT AAA GCA TAT GAG AGT GTA CTG and *reverse*: 5´-GGT TCA TTA TTC GGG CAA TTA C; IL-15 *forward*: 5’-CCA TCT CGT GCT ACT TGT GTT TCC and *reverse*: 5’-TCC AGT TGG CCT CTG TTT TAG G. The optimal primer concentrations were found to be 500 nM with all primers reaching an efficiency >99%. Forty cycles of amplification were performed (45 seconds 95°C, 1 minute 60°C), and a dissociation curve analysis confirmed the amplification of a single band. Mean CT values were obtained from duplicate reactions and relative abundances of transcripts in a given sample were first calculated as difference in CT (ΔCT) compared to MLN51 (*forward*: 5’-CCA AGC CAG CCT TCA TTC TTG and *reverse*: 5’-TAA CGC TTA GCT CGA CCA CTC TG for SYRB and *probe*: 5’ CAC GGG AAC TTC GAG GTG TGC CTA AC for TaqMan detection, respectively), the most stable housekeeping gene in lymphoid tissues [38]. Finally, fold expression change was calculated relative to WT (2^-ΔΔCT^).

### Isolation of splenic stroma

Splenic tissue was transferred into a petri dish containing a nylon strainer and carefully crushed with a glass-punch to remove lymphocytes. The remaining stroma tissue was washed lysed (in 200μl of 2D-lysisbuffer, pH 8.5, containing 7 M urea, 2 M thiourea, 4% (w/v) CHAPS and 30 mM Tris). The lysates were sonified on ice using a sonifier microtip at 20% amplitude (Branson Sonifier 250, Danburg, CT, USA), centrifuged (25,000 x g for 20 min at 4°C), and the supernatant was used for further analysis.

### 2D difference gel electrophoresis (2D-DIGE)

2D DIGE was performed as described [39]. In brief, the protein content of individual stroma cell lysates was defined by a Coomassie protein assay kit (Thermo, Rockford, IL, USA). Fluorescent labeling was performed with 50 μg protein lysate of each sample and 400 pmol of Cy5 or Cy3 (GE Healthcare) on ice for 30 min in the dark. The reaction was quenched by adding 1 μl lysine (10 mM) for 10 min. In addition, a mixture of all samples was labeled with Cy2 using as an internal control for inter-gel comparison. Labeled samples (50 μg each of Cy5, Cy3 and Cy2) were mixed with 100 μg unlabeled pooled protein. A total of 250 μg protein in 250 μl 2D-lysisbuffer were further mixed with 1% (v/v) IPG buffer pH 3–11 and 1% (w/v) DTT. The mixed samples were applied by cup loading onto 24 cm pH 3–11 non-linear IPG strips, rehydrated in DeStreak solution (GE Healthcare). Isoelectric focusing (IEF) was carried out for a total of 68 kVh with gradual increase of voltage (150 V for 3 h, 300 V for 3 h, 500 V for 3 h, gradient from 500 V to 1000 V for 6 h, gradient from 1000 to 10000 V for 4 h and 10000 for ~4 h). IPG strips were further reduced (0.5% (w/v) DTT) and alkylated (4.5% (w/v) iodoacetamide) in SDS-equilibrium buffer (6 M urea, 50 mM Tris-HCl, pH 8.8, 30% glycerol, 2% SDS, trace of bromophenol blue) and subsequently subjected to the second dimension on 12.5% polyacrylamide gels using the Ettan Dalt six system (GE Healthcare) at 12 mA per gel and continuous cooling at 20°C.

### Image analysis and spot picking

The in-gel fluorescence of different experimental setups were detected at three different emission wavelengths (Cy5: 670 mm, Cy3: 580 nm, Cy2: 520 nm) using a Typhoon Trio (GE Healthcare). Spots in the multiplexed gel images were detected by differential in-gel analysis (DIA) included in the DeCyder 6.5 software package (GE Healthcare) according to the manufactures instructions. Detection of spot boundaries followed by normalization of spot volumes revealed regulated proteins. For biological variance analysis (BVA) Cy2 images were merged resulting in vectors used for Cy5 and Cy3 image alignment. Quantification of each matched spot was accomplished by comparing the spot volumes ratios of their respective Cy5 and Cy3 fluorescence in the biological replicates. The level of significance among the replicates was compiled by student’s t-test set to 0.05. Since we and others observed molecular weight shifts resulting by minimal labeling [40], gels were post-stained with Flamingo Pink (Bio Rad) according to the manufactures instructions. The post-stained gels were analyzed using Image Master 6.0 (GE Healthcare). Respective differential spots resulting from the BVA were selected and picked by Ettan spotpicker (GE Healthcare) equipped with a 2 mm picking head according to the manufacturer’s instructions. Corrected punching of the spots was verified by rescanning.

### Proteolytic cleavage and mass spectrometric analysis

The tryptic digestion and mass spectrometric analysis was performed as described before [41]. In brief, after washing gel pieces in 25 mM ammonium bicarbonate (ABC) and shrinking them in pure ACN, they were rehydrated with 100 ng porcine trypsin (Serva, Heidelberg, Germany) in 10 μl of 5 mM ABC. After overnight digestion at 37°C and subsequent sonification in a water bath, liquid phases were collected, dried, redissolved in 0.8 μl α-cyanohydroxycinnamic acid (3.2 mg/ml, Sigma) in 65% ACN / 0.1% TFA and spotted onto 192-well stainless steel MALDI plate and air-dried. Spotted peptides were analyzed by MALDI-tof/tof mass spectrometry using a 4700 Proteomics Analyzer, calibrated with 4700 Calibration mix (Applied Biosystems, Framingham, MA, USA). Peptide mass spectra were processed by internal calibration with autolytic fragments of porcine trypsin with 25 ppm mass tolerance. Default calibration, updated prior the run, was used to acquire MSMS spectra with a fragment tolerance of 0.2 Da. MS and MSMS data were submitted to a MASCOT (version 2.0) search for identification using GPS Explorer 3.0 software. Entries of the NCBI non-redundant database for *Mus musculus* were searched with following parameters: fixed carbamidomethylation of cysteine residues, variable methionine oxidation; one missed trypsin cleavage; the monoisotopic masses were considered and with a mass accuracy of 50 ppm. A protein was considered identified with a probability of greater 95% if the MASCOT score was over 66.

### Western blotting

Western blotting was performed on 4–12% gradient Bis-Tris gels (Invitrogen, Karlsruhe, Germany) using 10 μg of splenic stroma lysates [41]. After electrophoresis proteins were transferred to 0.2 μm nitrocellulose membranes (Biometra, Goettingen, Germany) and subsequently blocked with bovine serum albumin in TBST (TBS + 0.05% Tween). The blots were analyzed with biotinylated anti-MFAP4 antibody (Hyb7-14, Odense, Denmark) and horseradish peroxidase coupled streptavidin. Anti-β-actin mAb (clone AC-15, Sigma) was used as loading control. Chemiluminescent detection was performed using ECL (enhanced chemiluminescence) reagents and Hyper Film (GE Healthcare). For densitometric analysis the blots were scanned in transmissive mode with the Molecular Imager GS-800 (Bio-Rad) and evaluated using the accompanying software Quantity One (version 4.6).

### Statistics

The statistics were performed with SPSS Statistics (IBM, Version 22). Differences between three or more groups were detected by using the Kruskal-Wallis-Test and for comparison of non-coherent data sets the Mann-Whitney-U-Test was conducted. Graphs were designed with SigmaPlot (Systat Software, San Jose, California, USA) and GraphPad Prism 5.0 (GraphPad Software Inc., La Jolla, USA).

## Results

### Growth of regenerating splenic tissue is suppressed by LTβR expression at non-splenic sites

**Fig 1:**
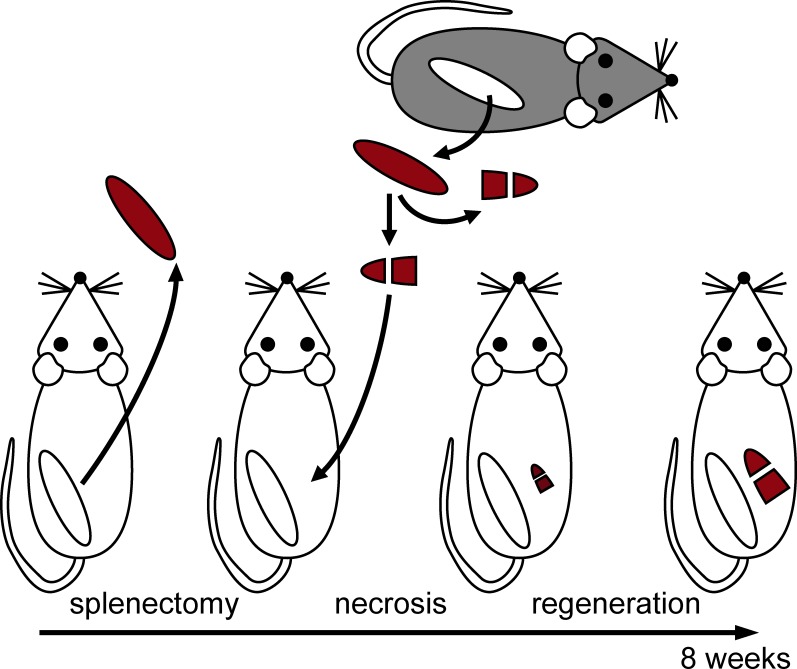
The splenic transplantation model. After removal of the endogenous spleen the recipient (WT or LTβR^-/-^) receives a splenic implant which is composed of two pieces representing 50% of a WT or LTβR deficient spleen. The implanted splenic tissue first becomes necrotic because the blood supply is lacking. Then, formation of splenic tissue starts and within eight weeks splenic regeneration is completed.

To analyze the impact of LTβR signaling within non-splenic tissues on the regeneration of splenic tissue we performed splenic tissue transplantation (Fig 1). Here, splenic tissue is implanted into the greater omentum of the peritoneal cavity after removal of the endogenous spleen (splenectomy). The implanted splenic tissue initially becomes necrotic because the blood supply is lacking. Then, formation of splenic lymphoid tissue starts and within eight weeks reproduces an equivalent to that of the adult organ. The splenic regenerate now reveals the four tissue compartments of a normal spleen (red pulp, marginal zone, B-cell zone, and T-cell zone) and performs typical splenic functions (e.g. removal of red blood cells, germinal center formation, clearance of blood borne pathogens [32, 33]).

**Fig 2:**
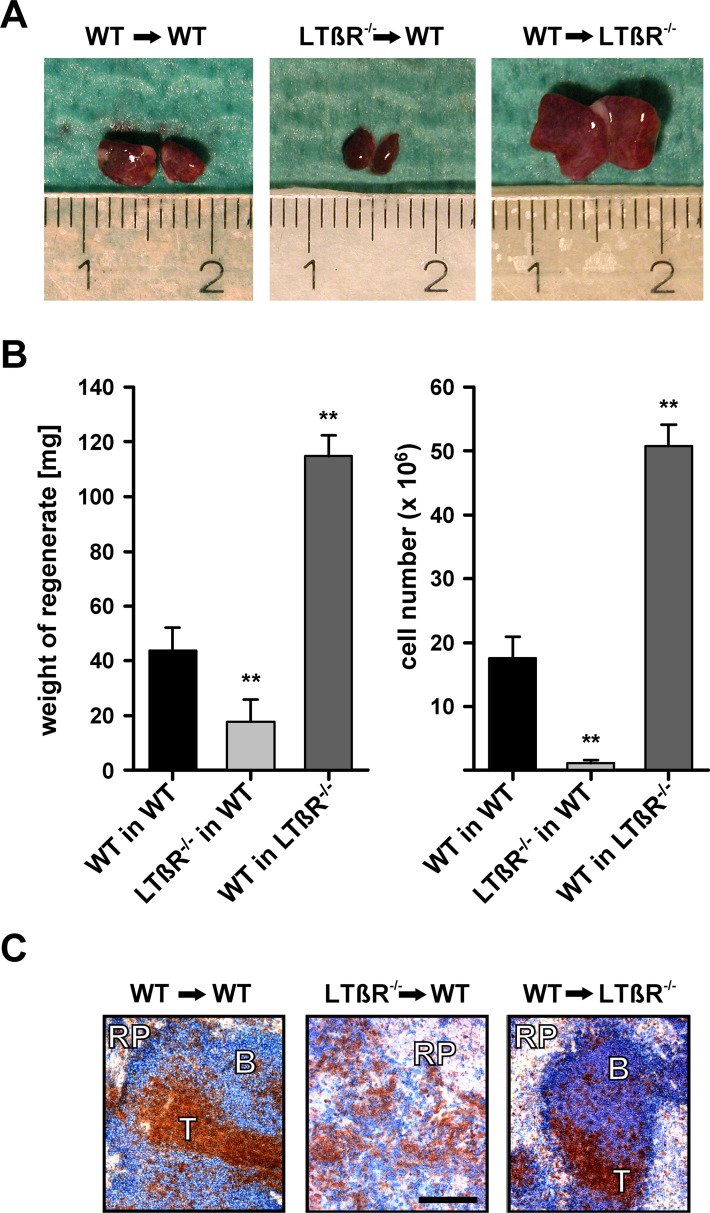
LTβR expression by non-splenic tissues suppresses growth of regenerating splenic tissue. (A) Macroscopic appearance of splenic regenerates 8 weeks after implantation of wild-type splenic tissue (WT) into wild-type recipients (WT), LTβR deficient splenic tissue (LTβR^-/-^) into WT recipients, and WT splenic tissue into LTβR deficient recipients (LTβR^-/-^). (B) Weight (left side) and cell number (right side) of splenic regenerates. Indicated are means and standard deviation (n = 4–9, ** = p < 0.01). (C) Microscopic appearance of splenic regenerates. Cryostat sections were stained by immunohistochemistry for T cells (brown; TCRβ^+^) and B cells (blue; B220^+^). Red pulp (RP), T-cell zone (T), and B-cell zone (B) are well developed except in the LTβR^-/-^ into WT combination where T and B cells are intermixed and only the red pulp (RP) is clearly recognizable (bar: 100 μm). This experiment was 3 times independently performed.

**Fig 3:**
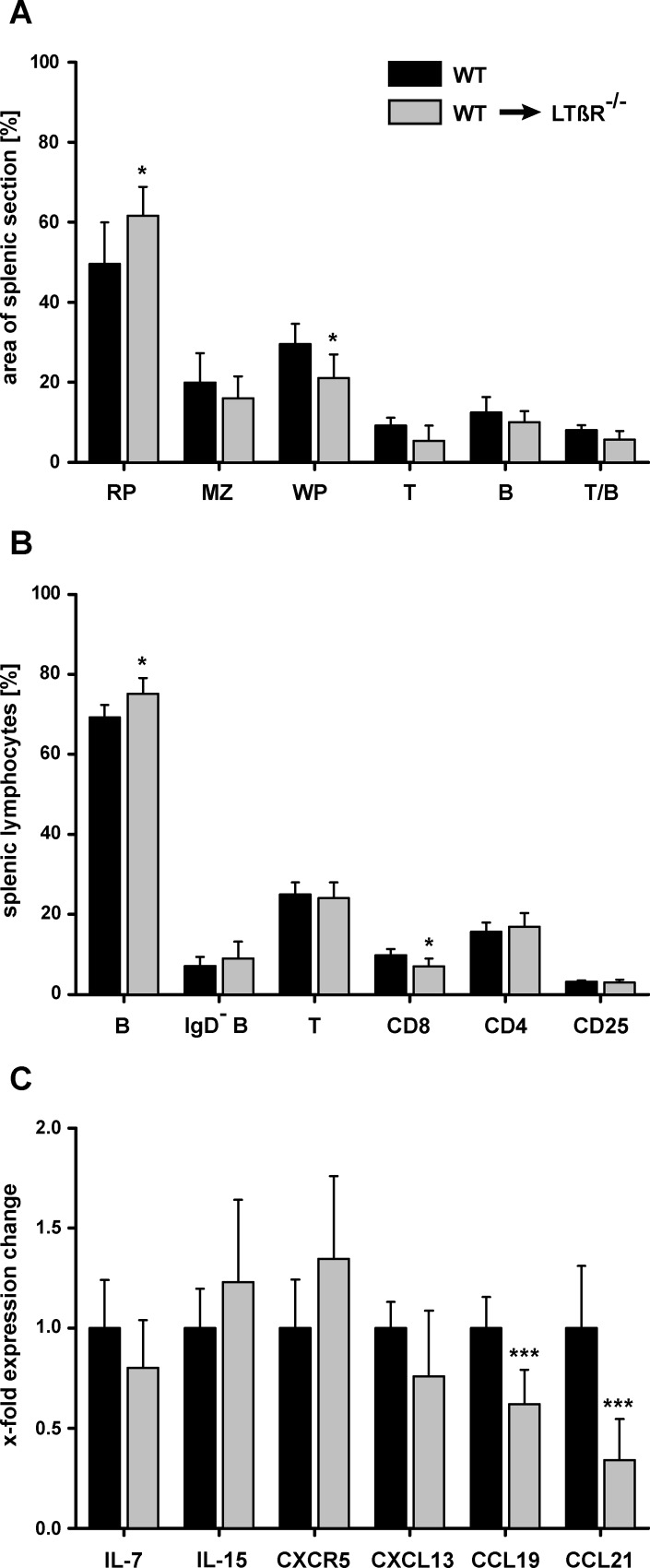
Architecture and cellular structure of WT spleen and WT into LTβR^-/-^ splenic regenerates are comparable. (A) The compartment sizes of normal WT spleens were analyzed and compared to that of splenic regenerates (WT→LTβR^-/-^) 8 weeks after implantation. Indicated is the size of individual splenic zones as percent of total section area (n = 6–7 animals per group; RP: red pulp, MZ: marginal zone, WP: white pulp, T: T-cell zone, B: B-cell zone, T/B: mixed T/B-cell zone). (B) Frequency of leukocyte subsets in normal WT spleens and splenic regenerates (WT→LTβR^-/-^) as determined by flow cytometry (n = 5–8 animals per group; B: B cells, IgD^-^ B: memory B cells, T: T cells; CD8: CD8^+^ T cells, CD4: CD4^+^ T cells, CD25: CD25^+^CD4^+^ -regulatory- T cells). (C) Expression of cytokines and chemokines in normal WT spleens and splenic regenerates (WT→ LTβR^-/-^). Shown is the relative expression level normalized to the housekeeping gene (MLN 51) and relative to WT (fold expression change). The bars represent the mean values ± SD. Mann-Whitney *U* test was used to indicate a significant difference between WT spleens and splenic regenerates (*p < 0.05; ***p < 0.001).

Thus, eight weeks after implantation of wild-type (WT) splenic tissue into the greater omentum of the abdominal cavity of splenectomized WT mice (WT → WT) splenic regenerates developed (Fig 2A) with a weight of about 40 mg (Fig 2B, S1 File) and showed all compartments of a normal spleen: red pulp, marginal zone, B-cell zone, and T-cell zone (Fig 2C). In contrast, when splenic tissue of LTβR deficient mice (LTβR^-/-^) was implanted into WT mice (LTβR^-/-^ → WT), regenerate weight and cell numbers were significantly reduced (Fig 2). T and B cells were intermixed and did not form a clearly defined T-cell zone and B-cell zone. Of the splenic compartments only the red pulp could be identified (Fig 2C). Surprisingly, implantation of WT splenic tissue into LTβR deficient recipients (WT → LTβR^-/-^) gave rise to regenerates with significantly increased weight and cell numbers compared to WT → WT regenerates. Like WT → WT regenerates, they revealed a histological appearance comparable to normal splenic tissue (Fig 2C). In WT → LTβR^-/-^ splenic regenerates the size of the red pulp was increased, whereas the size of the marginal zone and that of the B- and T-cell zones was similar to that of a normal WT spleen (Fig 3A). In addition, no difference was seen for most B- and T-cell subsets (Fig 3B) and for the expression of IL-7, IL-15, CXCR5, and CXCL13. However, CCL19 and CCL21 were reduced (Fig 3C). RORγt which is preferentially expressed by lymphoid tissue inducer cells [42] could not be detected in normal WT spleens (7 out of 7) whereas a detectable amount of transcript was present in 4 out of 7 WT → LTβR^-/-^ splenic regenerates indicating that process of splenic tissue regeneration was still going on. Thus, LTβR expression outside the regenerating splenic tissue reduced the growth of splenic regenerates. If the growth suppressive activity was absent (as in LTβR deficient recipients) splenic regenerates developed resembling a normal spleen both in size and structure.

### The size of splenic regenerates is determined by the number of LTβR expressing cells present in distant tissues

**Fig 4:**
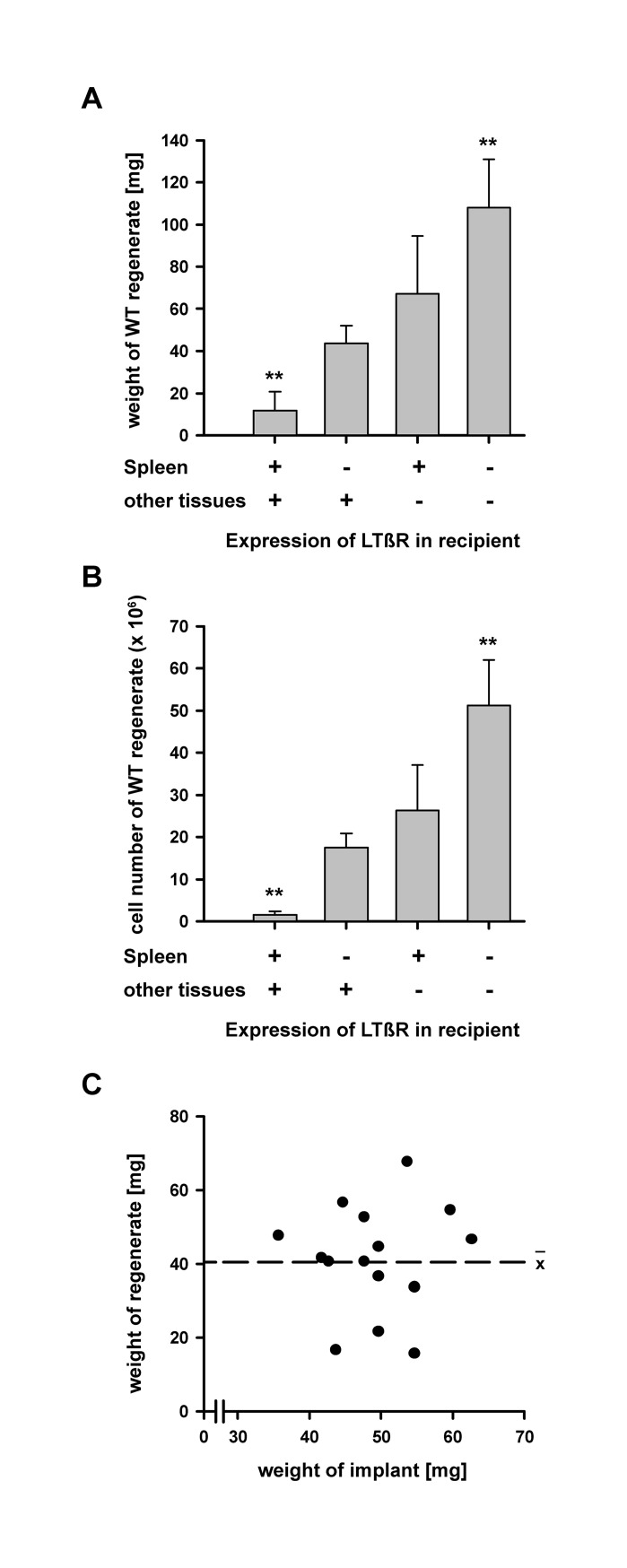
The final size of regenerating splenic tissue is determined by the number of LTβR expressing cells at distant sites. (A) Weight of splenic regenerates eight weeks of implantation into 4 types of recipients: i) WT mice that expressed LTβR in spleen (+) as well in other tissues (+); ii) splenectomized WT mice that expressed LTβR not in the spleen (-) but only in other tissues (+); iii) LTβR deficient mice that received WT splenic implants 8 weeks earlier, therefore expressing LTβR only within the regenerated splenic tissue (+) but not in other tissues (-); iv) LTβR deficient mice expressing LTβR neither in the spleen (-) nor in other tissues (-). Indicated are means and standard deviation (n = 5–9, ** = p < 0.01). (B) Cell number of splenic regenerates eight weeks of implantation into 4 types of recipients. Indicated are means and standard deviation (n = 5–9, ** = p < 0.01). (C) Indicated is the weight of the splenic implant and the weight of the resulting regenerate 8 weeks after implantation. There was no correlation between both parameters (Spearman-ρ = -0.1). The broken line indicates the mean. Each dot represents one animal. These experiments were 2 times independently performed.

To quantitate the suppressive activity of LTβR expression at distant sites on the regeneration of splenic tissue, WT splenic tissue was implanted into recipients with differential expression of LTβR: i) WT mice that expressed LTβR in spleen as well in non-splenic tissues; ii) splenectomized WT mice that expressed LTβR only in non-splenic tissues; iii) LTβR deficient mice that received WT splenic implants 8 weeks earlier, therefore expressing LTβR only within the regenerated splenic tissue; iv) LTβR deficient mice expressing LTβR neither in the spleen nor in non-splenic tissues. WT splenic tissue was then implanted into the recipients of the four experimental groups and analyzed 8 weeks later. Our results showed that the parallel expression of LTβR in spleen and non-splenic tissues (normal WT mice) exerted maximum suppression of implant regeneration regarding weight and cell numbers (Fig 4A and 4B). Removal of the spleen (splenectomized WT mice) significantly relaxed the suppressive activity of LTβR expression (Fig 4A and 4B). Complementary observation was made in animals expressing LTβR in regenerated splenic tissue but not in non-splenic tissues (LTβR deficient mice with WT regenerate). The parallel lack of LTβR expression in spleen and in non-splenic tissues (LTβR deficient mice) exerted the least suppressive activity and the splenic regenerates became exceedingly large (Fig 4A and 4B). These results demonstrated that weight and cell number of splenic regenerates negatively correlate with the number of LTβR expressing cells within the whole body.

To analyze whether the final size of regenerating splenic tissue is mainly determined by the number of LTβR expressing cells within or outside the implanted splenic tissue, WT splenic implants of different weight were implanted into splenectomized WT recipients and the initial weight of the implant was compared to the weight of the regenerate. There was no correlation between the input weight of splenic implants and the output weight of splenic regenerates (Fig 4C). This suggests that the final size of splenic regenerates is not primarily determined by the number of LTβR expressing cells within the regenerating tissue, because in this case a correlation between input and output weight would have been expected. Instead, independent of the input weight, the final weight of splenic regenerates ranged around 40 mg which strongly indicates that the number of LTβR expressing cells within non-splenic tissues is the size determining factor.

Taken together, the present data demonstrate that the number of LTβR expressing cells in splenic and non-splenic tissues inversely correlates with the size of splenic regenerates. Thus, the LTβR dependent growth suppressive activity seems to determine the weight up to which splenic tissue can grow.

### LTβR dependent growth suppressive activity affects fully developed splenic tissue and is not executed via LTβR signaling

**Fig 5:**
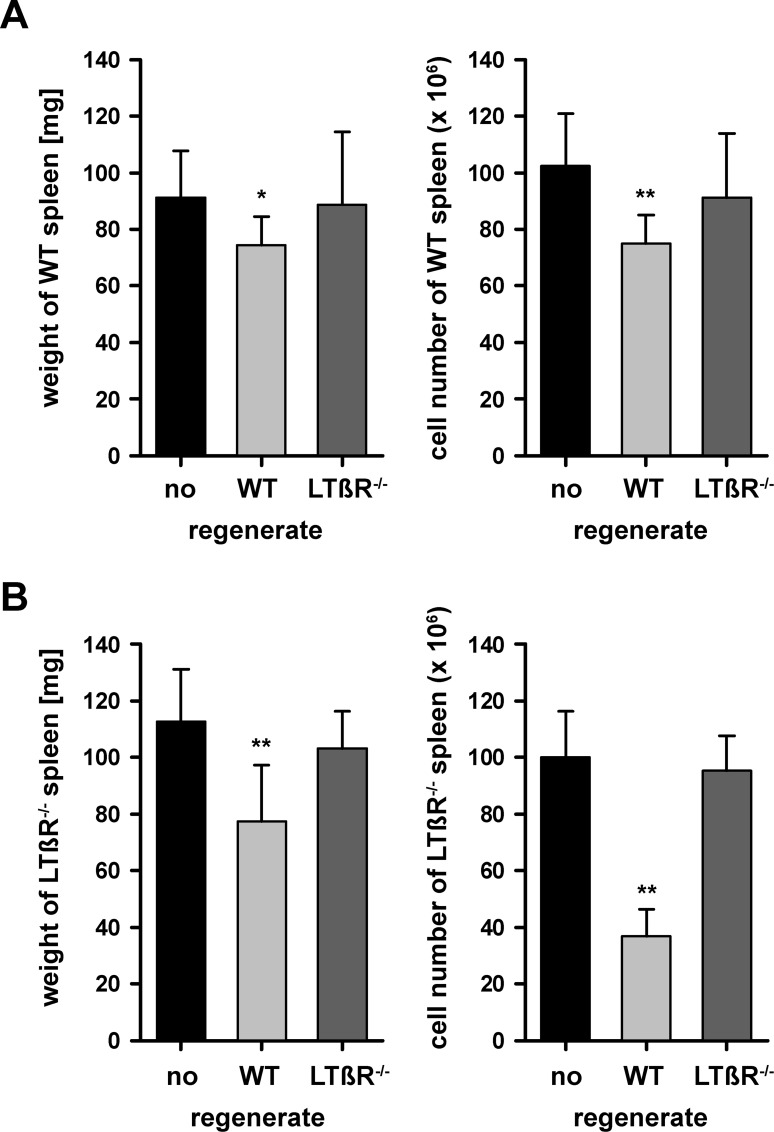
LTβR dependent growth suppressive activity also affects fully developed splenic tissue. (A) WT recipients without prior splenectomy were either sham operated or received WT or LTβR^-/-^ splenic implants. Eight weeks later weight (left side) and cell number (right side) of the endogenous spleen was determined. Only WT splenic regenerates significantly reduced weight and cell number of the endogenous WT spleen. Indicated are means and standard deviation (n = 4–11, * = p < 0.05, ** = p < 0.01). (B) LTβR^-/-^ recipients without prior splenectomy were either sham operated or received WT or LTβR^-/-^ splenic implants. Eight weeks later weight (left side) and cell number (right side) of the endogenous spleen was determined. Only WT splenic regenerates significantly reduced weight and cell number of the endogenous LTβR^-/-^ deficient spleen. Indicated are means and standard deviation (n = 5–8, ** = p < 0.01). These experiments were 2 times independently performed.

To investigate whether not only the size of regenerating splenic tissue but also that of a fully developed spleen is affected by the LTβR dependent suppressive activity, WT splenic tissue was implanted into WT mice with their endogenous spleen preserved and 8 weeks later weight and cell number of the endogenous spleen were determined. Both weight and cell numbers of the endogenous spleen were significantly reduced by the splenic regenerate (Fig 5A). This growth suppressive activity was due to the LTβR expression of the splenic regenerate, since it did not occur when LTβR deficient splenic tissue was implanted (Fig 5A). To exclude redistribution of cells from the endogenous spleen into the splenic regenerate as possible cause for the observed reduction in weight and cell number of the endogenous spleen, we determined the cell number in normal spleens and compared it to the combined cell number of the endogenous spleen and the splenic regenerate. Normal spleens contained 102 ± 10 (x10^6^; n = 11) cells whereas the combined cell number of endogenous spleen and regenerated splenic tissue significantly decreased to 77 ± 11 (x10^6^; n = 5; p<0.05). This observation excludes redistribution of cells as a possible cause for the LTβR dependent size reduction of the endogenous spleen.

Next it was investigated whether the LTβR dependent suppressive activity is not only induced but also executed via the LTβR pathway. Therefore, WT splenic tissue was implanted into LTβR deficient recipients with their endogenous spleen preserved and it was examined whether weight and cell number of the endogenous spleen would be reduced despite the lack of LTβR expression. Indeed, under the influence of a WT splenic regenerate, both weight and cell numbers of the LTβR deficient endogenous spleen were significantly reduced (Fig 5B). This reduction was not observed, if LTβR deficient splenic implants were used (Fig 5B). This shows that the LTβR dependent suppressive activity is not executed via LTβR expressed within splenic tissue.

### Molecular characterization of the LTβR dependent growth suppressive activity

**Fig 6:**
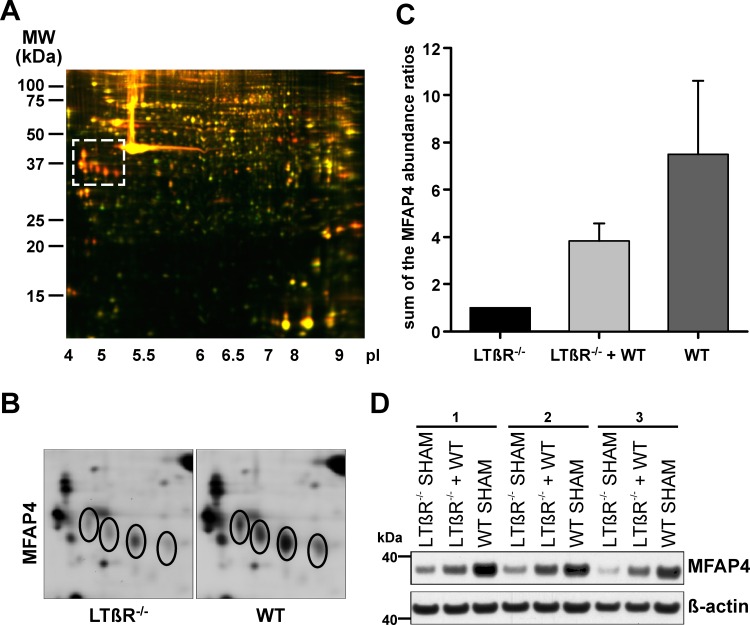
Identification of LTβR regulated proteins by two-dimensional differential gel electrophoresis and mass spectrometry. (A) 2D-DIGE experiment performed with splenic stroma of LTβR^-/-^ (Cy3, green) and WT (Cy5, red) mice. The Cy2 channel is masked (representative gel of three replicas). The encircled area is shown in (B). (B) Gel section showing four spots with an increase in volume ratio when LTβR^-/-^ (left side) and WT (right side) splenic stroma was compared. Mass spectrometry identified the four spots as four isoforms of MFAP4 (representative gel of three replicas). (C) Indicated is the MFAP4 abundance in splenic stroma of LTβR^-/-^ spleen, LTβR^-/-^ spleen from a donor which received a WT splenic implant 8 weeks earlier (LTβR^-/-^ + WT), and WT spleen. The different 2D-DIGE experiments were compared quantitatively by summing up the volume ratios of the four isoforms of MFAP4 and relating it to the abundance of MFAP4 in LTβR^-/-^ splenic stroma which was set to one. The abundance of MFAP4 increased from LTβR^-/-^ splenic stroma to LTβR^-/-^ splenic stroma obtained from mice with a WT splenic regenerate and further to WT splenic stroma. Indicated are means and standard deviation (n = 3–6). This experiment was 2 times independently performed. (D) Western blot analysis of MFAP4 from splenic stroma lysates of LTβR^-/-^ mice, LTβR^-/-^ mice which received a WT splenic implant 8 weeks earlier (LTβR^-/-^ + WT), and WT mice. Three different sets of experiments are shown.

Since it is known that LTβR is not expressed by lymphocytes [17], in the subsequent experiments splenic stroma was studied. A quantitative proteome analysis was carried out to perform an unbiased search for other candidate factors. Two-dimensional differential gel electrophoresis (2D-DIGE) was performed on splenic stroma of LTβR deficient and WT mice (n = 3 pairs) and the proteomes were compared. To increase the specificity of our approach, we also compared the proteomes of splenic stroma from LTβR deficient mice without or with a splenic WT regenerate (n = 3). In this situation the WT regenerate reduces the size of the endogenous spleen (Fig 5). Thus, an inhibitory factor produced by the WT regenerate would be detectable within the stroma of the endogenous LTβR deficient spleen leading to a higher abundance compared to that of splenic stroma of LTβR deficient spleen from a donor without WT regenerate. The subsequently detected differential spots (over 2000 per pair) were selected when they were present in all samples and showed a change in the average volume ratio of >2.0 or <-2.0 (p <0.05). Appling these criteria we detected 61 proteins, 22 occurring in higher and 39 in lower abundance in WT splenic stroma and splenic stroma of LTβR deficient spleen from donors with a splenic WT regenerate. Mass spectrometric analysis showed that primarily microfibrillar-associated protein 4 (MFAP4) impressed in both sets of experiments by its high abundance and significant volume ratio change (Fig 6A). It appeared in four isoforms and each of them increased in its volume ratio 2.3 to 2.8-fold when LTβR deficient splenic stroma was compared to WT splenic stroma (Fig 6B). In order to compare the different 2D-DIGE setups quantitatively, the sum of the volume ratios was normalized to the abundance in LTβR deficient mice (that was set to one) clearly showing that the abundance of MFAP4 isoforms increased from LTβR deficient splenic stroma to LTβR deficient splenic stroma obtained from mice with a WT splenic regenerate and even further to WT splenic stroma (Fig 6C). This pattern was confirmed by Western blot analysis, demonstrating an about 7 fold difference in MFAP4 abundance between LTβR deficient and WT splenic stroma (Fig 6D). Our approach shows that MFAP4 is up-regulated at the protein level by the LTβR pathway. Furthermore, we demonstrate that MFAP4 produced at distant sites (WT splenic regenerate of a LTβR deficient recipient) is imported into the endogenous spleen of those recipients.

### Functional characterization of the LTβR dependent growth suppressive activity

In a first attempt to characterize the biological function of the LTβR dependent suppressive activity we concentrated on MFAP4 as possible candidate because of three reasons. First, thymic implants show much less growth restriction than splenic implants [43, 44] which coincides with down-regulated MFAP4 mRNA levels in the thymus [45]. Second, MFAP4 blood levels significantly correlate with liver fibrosis [46] suggesting that MFAP4 might interfere with tissue regeneration. Third, an absolute requirement for the LTβR dependent inhibitory factor is its ability to reach splenic tissue after being produced by non-splenic sites which has recently been demonstrated for MFAP4 [47].

**Fig 7:**
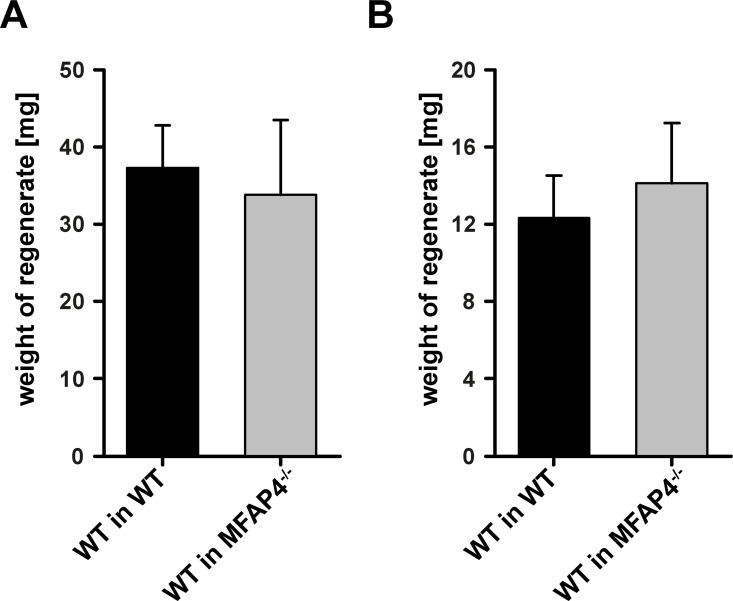
MFAP4 does not suppress the regeneration of splenic implants. (A) WT and MFAP4 deficient recipients were splenectomized and then received a WT splenic implant. Eight weeks later the weight of the splenic regenerate was determined. No difference was seen between WT and MFAP4 deficient recipients. Indicated are means and standard deviation (n = 6). (B) WT and MFAP4 deficient recipients without prior splenectomy received a WT splenic implant. Eight weeks later the weight of the splenic regenerate was determined. No difference was seen between WT and MFAP4 deficient recipients. In addition, the suppressive activity of the endogenous spleen was similar in both groups (compare weights to the weights under A). Indicated are means and standard deviation (n = 6). These experiments were 2 times independently performed.

To test the biological function of MFAP4 we implanted WT splenic tissue into WT and MFAP4 deficient recipients. The expectation was that splenic implants would grow to significantly larger regenerates in MFAP4 deficient recipients. However, no difference was seen when WT tissue was implanted into splenectomized WT or MFAP4 deficient mice (Fig 7A). In addition, we could also not demonstrate a difference in the suppressive capacity of the endogenous spleen on the splenic regenerate which we expected to be less in the presence of MFAP4 deficient spleen (Fig 7B). Instead, our results confirmed the suppressive effect of the endogenous spleen on regenerating splenic tissue for both WT and MFAP4 deficient animals (weight of splenic regenerate: about 10 mg with endogenous spleen and 40 mg without, Fig 7A and 7B).

## Discussion

LTβR expression is essential for normal splenic development during ontogeny [25] and for regeneration of splenic tissue in adult life [48, 33]. In the present study we confirm these observations by showing that implantation of LTβR deficient splenic tissue into WT mice results in splenic regenerates that, compared to WT implants, show significantly reduced weight and cell numbers together with poorly developed B- and T-cell zones and an absent marginal zone (Fig 2). It is known that cells of the recipient are able to enter regenerating splenic tissue in small numbers [47] possibly ferrying LTβR expression into LTβR deficient splenic tissue. However, our study shows that this occurs at a functionally irrelevant level since the implantation of LTβR deficient splenic tissue into either WT (import in principle possible; present study) or LTβR deficient recipients (import impossible; [33]) leads to a comparably poor regeneration. This demonstrates that regeneration of splenic tissue strictly depends on endogenous stromal cells thereby confirming earlier observations [32]. In contrast, regeneration of WT splenic tissue is tremendously enhanced in the absence of LTβR expression in the recipient. Weight and cell numbers are significantly higher than after implantation into a WT recipient. In addition, a marginal zone is present, B-cell and T-cell zones are well developed, and the composition of B- and T-cell subsets is similar to that of a normal spleen (Fig 3). The reduced expression of CCL19 and CCL21 and the presence of lymphoid tissue inducer cells (as indicated by RORγt expression) in 4 out of 7 of WT → LTβR^-/-^ splenic regenerates suggests that these large regenerates (120mg) need more time for completing the process of regeneration than the WT → WT splenic regenerates (40mg) for which 8 weeks have been determined [33]. Our results demonstrate that LTβR expressed outside the regenerating tissue is able to suppress the growth of splenic implants, whereas in its absence splenic regenerates develop which are similar to a normal spleen in size, structure and cellular composition.

This growth suppressive activity is induced both by LTβR expressed in the spleen (increased growth of splenic regenerates in splenectomized WT recipients) and in non-splenic tissues (even more growth in LTβR deficient recipients). It is very likely that LTβR expressed in lymph nodes contributes to the suppressive activity of non-splenic tissues as mice which lack all lymph nodes have spleens that are significantly enlarged compared to control spleens [49, 50]. Our data further indicate that the LTβR dependent growth suppressive activity induced by the spleen and non-splenic tissues is comparable (Fig 4). This suggests that the growth suppressive activity does not depend on lymphocyte numbers because the spleen contains only half the number of lymphocytes found in all lymph nodes together [51]. In addition, the LTβR dependent growth suppressive activity not only affects regenerating splenic tissue but also the fully developed spleen (Fig 5). The growth suppressive activity of the LTβR signaling pathway is considerable. In its full presence splenic implants are only allowed to form regenerates of 10 mg weight with poor structure (Fig 4B, left bar), whereas in its absence it is more than 10 times that weight with excellent morphology (Fig 3; Fig 4B, right bar). The LTβR dependent growth suppressive activity correlates with the number of receptor expressing cells outside the regenerating tissue being highest in wild type recipients, lowest in LTβR deficient recipients, and in between when either spleen or non-splenic tissues (e.g. lymph nodes) express LTβR (Fig 4).

Thus, in addition to its essential role in development and maintenance of secondary lymphoid tissues [24], the present study demonstrates for the first time that LTβR expressed at distant sites exerts a growth suppressive activity on developing and developed splenic tissue. The two features of the LTβR signaling pathway—supportive and suppressive—makes it an ideal candidate for regulating the size of splenic tissue. If this conclusion is correct than the size of splenic regenerates should not primarily depend on the amount of tissue implanted (and thus the number of LTβR expressing cells). Instead, the number of LTβR expressing cells at distant sites should be involved in determining the final size of splenic regenerates. And indeed, splenic regenerates reach a threshold weight that does not correlate with the amount of implanted tissue but depends on the total number of LTβRs expressed in the recipient (Fig 4C). Lack of correlation between the weight of implanted and regenerated splenic tissue is also seen in rat, pig, sheep and man [32] suggesting that the mechanisms observed in the present study for mice may also be operative in other species. Future studies will analyze i) whether LTβR expressed by secondary lymphoid tissues other than spleen and lymph nodes (e.g. Peyer’s patches and tonsils) also suppress splenic growth, ii) whether the growth suppression exerted by LTβR expressed within lymphoid tissues is different from that exerted by LTβR expressed within non-lymphoid tissues, iii) and whether a possible difference in LTβR signaling of lymphoid and non-lymphoid tissues is linked to different ligand usage (LTα_1_β_2_, LIGHT) or the alternative and classical pathway, respectively [52, 23]. Furthermore, it seems very likely that the growth suppressive activity of LTβR not only affects splenic tissue but also lymph nodes, since after splenectomy the weight of mesenteric lymph nodes increases in rats (control: 130 ± 20 mg, n = 6; splenectomized: 170 ± 20 mg, n = 6; own unpublished observations). Taken together, the dual role of the LTβR (supporting and suppressing growth of lymphoid tissue) makes this signaling pathway an ideal candidate for regulating the total size of secondary lymphoid organs within narrow limits during homeostasis. During infections, however, lymph node size increases up to 10 fold within 5 days [53, 54] and in the course of autoimmune diseases tertiary lymphoid tissue of considerable size may develop [55, 56, 57]. This indicates that the regulatory role of LTβR signaling regarding lymphoid tissue size probably differs during homeostasis and immune responses, although LTβR signaling is essential for both processes [58, 59, 23, 60].

The LTβR dependent growth suppressive activity could be executed in principle either by inhibition of growth promoting factors or induction of growth inhibiting factors. Our study provides evidence for the latter. Implantation of WT splenic tissue into LTβR deficient recipients leads to huge and well-structured regenerates (115 mg) which is compatible with both scenarios (availability of growth promoting factor or absence of growth inhibiting factor). However, implantation of a second WT splenic implant leads to splenic regenerates that are significantly smaller than the first one (65 mg, Fig 4). This observation is only compatible with the hypothesis that the first WT regenerate produces growth inhibiting factors, but not with the assumption that the LTβR deficient recipients continues to produce growth promoting factors. The presence of LTβR induced growth suppressing factors is also supported by a study in which mice were treated for one month with LTβR antagonists to block LTβR signaling [61]. The authors report that splenic cell numbers increase from 60 to 140 x10^6^ suggesting that blockage of LTβR signaling also blocked the production of growth suppressing factors.

In a first attempt to characterize the inhibitory factor at the molecular level, we concentrated on microfibrillar-associated protein 4 (MFAP4) which is an oligomeric extracellular protein belonging to the fibrinogen-related domain protein superfamily [62, 63, 64] and binds to extracellular matrix fibrils in a calcium dependent manner [65]. This suggests that MFAP4 mediates cell-to-cell and cell-to-matrix interactions thereby contributing to maintenance of tissue integrity [62, 65, 63, 64]. In addition, MFAP4 blood levels significantly correlate with fibrosis of liver tissue [46] suggesting that MFAP4 might interfere with tissue regeneration in general and thus might inhibit splenic tissue growth. Furthermore, MFAP4 mRNA levels in the thymus are specifically down-regulated [45] which coincides with the observation that thymic implants show much less growth restriction than splenic implants [43, 44]. For example, by increasing the weight of implants the final weight of thymic regenerates in rats can reach 800 mg which is nearly 5 times the weight of a normal thymus [43, 44]. And indeed, we demonstrated at the protein level that MFAP4 expression is up-regulated by the LTβR pathway (Fig 6). However, when MFAP4 was tested in our transplantation model, we failed to see any effect on splenic tissue regeneration. Implantation of splenic WT tissue into splenectomized MFAP4 deficient mice does not lead to the enhanced regeneration which we expected to see (Fig 7). In addition, comparable to WT recipients, the endogenous spleen of MFAP4 deficient recipients does suppress the regeneration of splenic WT implants. Thus, splenic transplantation is a useful method to determine the biological function of candidate factors. At the moment we broaden the selection criteria for candidate proteins which will be investigated by this approach. Finally, although the production of the growth inhibitory factor(s) is induced via the LTβR signaling pathway, their activity is not executed via this pathway because both WT and LTβR deficient splenic tissues are sensitive to the growth inhibitory factor(s) (Fig 5).

### Perspective

**Fig 8:**
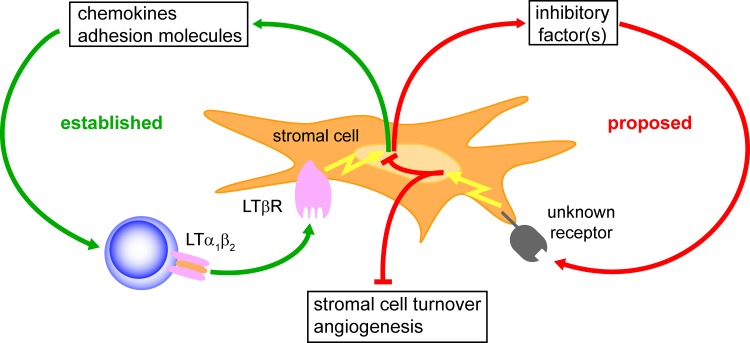
The two faces of the LTβR signaling pathway. After LTα_1_β_2_ expressed by activated B and T cell binds to LTβR, stromal cells up-regulate the secretion of chemokines (e.g. CCL19, CCL21, CXCL13) and the expression of adhesion molecules (e.g. ICAM-1, VCAM-1, MAdCAM-1). This further attracts LTα_1_β_2_ expressing cells and establishes a positive feed-back loop that is essential for the development and maintenance of secondary lymphoid organs (left side, green). The present study now indicates that ligation of the LTβR also leads to production of growth inhibitory factor(s) which upon binding to unknown receptors expressed by not yet characterized cells might counteract the positive feed-back loop. Interference with stromal cell turnover and/or angiogenesis might be possible targets (right side, red). When reaching a threshold level, LTβR dependent supportive and suppressive activities are in balance and secondary lymphoid organs homeostasis is achieved.

It is well established that the presence of LTβR inside splenic tissue is essential for supporting its development during ontogeny and its maintenance during adult life (Fig 8, left side, green). The present study now suggests that a growth suppressive activity is also exerted by LTβR expressed both in splenic and in non-splenic tissues. We provide evidence for the LTβR dependent production of growth inhibitory factor(s) most likely by stromal cells (Fig 7, right side, red). The two faces of LTβR expression (support and suppression of growth) might represent a molecular mechanism for regulating the final size of secondary lymphoid organs during development and homeostasis. Thus, the biology of LTβR signaling can only be fully appreciated if its expression inside and outside the tissue of interest is considered. In addition, the two faces of LTβR signaling should also be taken into account when the LTβR pathway is therapeutically targeted [16, 11]. For example, when mice are temporarily treated with a LTβR antagonist, lymph node cell numbers decrease by 10 x 10^6^ (i.e. blocking the supportive effect) whereas at the same time spleen cell numbers increase by 80 x 10^6^ (i.e. blocking the suppressive effect; [61]), an effect not seen when mice are continuously treated (i.e. blocking the supportive effect prevails [66]). In addition, after identification of the growth inhibitory factor(s) and their receptor(s) they might be used to eradicate tertiary lymphoid tissues that induce and maintain autoimmune and chronic inflammatory diseases [67, 56, 55, 68]. Furthermore, neutralization of the growth inhibitory factor(s) by specific antibodies might significantly increase the efficiency of vaccinations or support the regeneration of therapeutically damaged lymphoid tissues.

## Supporting Information

S1 File Data availability. This file contains the data presented in the Figs 2–7.
(DOCX)
