# Supplementary material for: Growth of Murine Splenic Tissue Is Suppressed by Lymphotoxin β-Receptor Signaling (LTβR) Originating from Splenic and Non-Splenic Tissues
Source: PLoS One. 2016 Dec 9;11(12):e0166901. doi: 10.1371/journal.pone.0166901 (PMC5147843; doi:10.1371/journal.pone.0166901)
Supplement: S1 File — This file contains the data presented in the Figs 2–7. (DOCX) [file pone.0166901.s001.docx]

Figure 2B Data Tables:

Weight of regenerate

| Group | WT in WT | LTßR^-/-^ in WT | WT in LTßR^-/-^ |
| --- | --- | --- | --- |
| Weight of regenerate [mg] | 44 | 18 | 115 |
| Standard Deviation [mg] | 8 | 8 | 8 |
| n | 9 | 6 | 4 |

Total number of leukocytes in the regenerate

| Group | WT in WT | LTßR^-/-^ in WT | WT in LTßR^-/-^ |
| --- | --- | --- | --- |
| Total number of leukocytes (x 10^6^) | 17.5 | 1.1 | 50.8 |
| Standard Deviation (x 10^6^) | 3.4 | 0.5 | 3.4 |
| n | 9 | 6 | 4 |

Figure 3A Data Table:

Single compartments proportion of total splenic section area [%]

|  | Group | | | | | |
| --- | --- | --- | --- | --- | --- | --- |
|  | WT | | | WT in LTßR^-/-^ | | |
| Compartment | Mean [%] | Std.Dev. | n | Mean [%] | Std.Dev. | n |
| Red Pulp (RP) | 49.5 | 10.4 | 6 | 61.6 | 7.2 | 6 |
| Marginal Zone (MZ) | 19.9 | 7.3 | 6 | 16.0 | 5.4 | 6 |
| White Pulp (WP) | 29.6 | 5.1 | 6 | 21.0 | 6.0 | 6 |
| PALS (T) | 9.2 | 1.9 | 6 | 5.4 | 3.8 | 6 |
| Folicle (B) | 12.4 | 3.9 | 6 | 10.0 | 2.8 | 6 |
| T/B mixed | 7.9 | 1.3 | 6 | 5.6 | 2.2 | 6 |

Figure 3B Data Table:

Subpopulations proportion of splenic lymphocytes [%]

|  | Group | | | | | |
| --- | --- | --- | --- | --- | --- | --- |
|  | WT | | | WT in LTßR^-/-^ | | |
| Subpopulation | Mean [%] | Std.Dev. | n | Mean [%] | Std.Dev. | n |
| B Lymphocytes (B) | 69.2 | 3.1 | 7 | 75.1 | 4.0 | 7 |
| IgD neg. B (IgD^-^ B) | 7.1 | 2.25 | 6 | 8.9 | 4.3 | 7 |
| T Lymphocytes (T) | 25.0 | 3.0 | 7 | 24.0 | 4.0 | 7 |
| CD8 pos. T (CD8) | 9.8 | 1.5 | 7 | 7.0 | 1.9 | 7 |
| CD4 pos. T (CD4) | 15.7 | 2.3 | 7 | 16.9 | 3.4 | 7 |
| CD25 pos. T (CD25) | 3.2 | 0.3 | 7 | 3.0 | 0.7 | 7 |

Figure 3C Data Table:

x-fold expression of cytokine/chmokine/receptor mRNA (WT set to 1)

|  | Group | | | | | |
| --- | --- | --- | --- | --- | --- | --- |
|  | WT | | | WT in LTßR^-/-^ | | |
| mRNA of | Mean [%] | Std.Dev. | n | Mean [%] | Std.Dev. | n |
| Interleukin-7 (IL-7) | 1.0 | 0.24 | 5 | 0.80 | 0.24 | 5 |
| Interleukin-15 (IL-15) | 1.0 | 0.20 | 5 | 1.23 | 0.41 | 5 |
| CXCR5 | 1.0 | 0.24 | 5 | 1.35 | 0.41 | 5 |
| CXCL13 | 1.0 | 0.13 | 5 | 0.76 | 0.33 | 5 |
| CCL19 | 1.0 | 0.16 | 5 | 0.62 | 0.17 | 5 |
| CCL21 | 1.0 | 0.31 | 5 | 0.34 | 0.21 | 5 |

Figure 4A Data Table:

Weight of WT regenerate

| Group | WT in WT (with endogenous spleen) | WT in WT (splenectomized) | WT in LTßR-/- (with former transplanted WT-spleen) | WT in LTßR^-/-^ (formerly untreated) |
| --- | --- | --- | --- | --- |
| Weight of regenerate [mg] | 12 | 44 | 67 | 108 |
| Standard Deviation [mg] | 9 | 8 | 27 | 23 |
| n | 6 | 9 | 8 | 8 |
| **LTßR expression in:** |  | | | |
| spleen | + | - | + | - |
| other tissues | + | + | - | - |

Figure 4B Data Table:

Total number of leukocytes in the WT regenerate

| Group | WT in WT (with endogenous spleen) | WT in WT (splenectomized) | WT in LTßR-/- (with former transplanted WT-spleen) | WT in LTßR^-/-^ (formerly untreated) |
| --- | --- | --- | --- | --- |
| Number of leukocytes (x 10^6^) | 1.3 | 17.5 | 26.3 | 51.2 |
| Standard Deviation (x 10^6^) | 1.0 | 3.4 | 10.7 | 10.9 |
| n | 6 | 9 | 8 | 8 |
| **LTßR expression in:** |  | | | |
| spleen | + | - | + | - |
| other tissues | + | + | - | - |

Figure 4C Data Table:

Weight of transplanted splenic tissue at day of Surgery and the corresponding weight of the regenerate 8 weeks later:

| Transplanted splenic tissue [mg] | Weight of regenerate [mg] |
| --- | --- |
| 36 | 47 |
| 63 | 46 |
| 42 | 41 |
| 48 | 52 |
| 50 | 44 |
| 54 | 67 |
| 48 | 40 |
| 43 | 40 |
| 55 | 15 |
| 50 | 36 |
| 60 | 54 |
| 55 | 33 |
| 45 | 56 |
| 44 | 16 |
| 50 | 21 |

Figure 5A Data Tables:

Weight of endogenous WT spleen

| Group | WT (no) | WT in WT (WT) | LTßR^-/-^ in WT (LTßR^-/-^) |
| --- | --- | --- | --- |
| Weight of endogenous spleen [mg] | 91 | 75 | 89 |
| Standard Deviation [mg] | 16 | 10 | 26 |
| n | 11 | 6 | 4 |

Total number of leukocytes in the endogenous WT spleen

| Group | WT (no) | WT in WT (WT) | LTßR^-/-^ in WT (LTßR^-/-^) |
| --- | --- | --- | --- |
| Number of leukocytes (x 10^6^) | 102.5 | 74.9 | 91.3 |
| Standard Deviation (x 10^6^) | 18.5 | 10.2 | 22.7 |
| n | 11 | 6 | 4 |

Figure 5B Data Tables:

Weight of the endogenous LTßR^-/-^ spleen

| Group | LTßR^-/-^ (no) | WT in LTßR^-/-^ (WT) | LTßR^-/-^ in LTßR^-/-^ (LTßR^-/-^) |
| --- | --- | --- | --- |
| Weight of endog. spleen [mg] | 91 | 75 | 89 |
| Standard Deviation [mg] | 16 | 10 | 26 |
| n | 11 | 6 | 4 |

Total number of leukocytes in the endogenous LTßR^-/-^ spleen

| Group | LTßR^-/-^ (no) | WT in LTßR^-/-^ (WT) | LTßR^-/-^ in LTßR^-/-^ (LTßR^-/-^) |
| --- | --- | --- | --- |
| Number of leukocytes (x 10^6^) | 102.5 | 74.9 | 91.3 |
| Standard Deviation (x 10^6^) | 18.5 | 10.2 | 22.7 |
| n | 11 | 6 | 4 |

Figure 6C Data Tables:

Sum of the MFAP4 abundance ratios (LTßR^-/-^ set to 1.0)

| Group | LTßR^-/-^ | LTßR^-/-^ + WT | WT |
| --- | --- | --- | --- |
| MFAP4 abundance ratio | 1.0 | 3.8 | 7.5 |
| Standard Deviation | 0.0 | 0.7 | 3.1 |
| n | 6 | 3 | 3 |

Figure 7A Data Table:

Weight of regenerate

| Group | WT in WT (splenectomized) | WT in MFAP4^-/-^ (splenectomized) |
| --- | --- | --- |
| Weight of regenerate [mg] | 37 | 34 |
| Standard Deviation [mg] | 5 | 10 |
| n | 6 | 6 |

Figure 7B Data Table:

Weight of regenerate

| Group | WT in WT (with endogenous spleen) | WT in MFAP4^-/-^ (with endogenous spleen) |
| --- | --- | --- |
| Weight of regenerate [mg] | 12 | 14 |
| Standard Deviation [mg] | 2 | 3 |
| n | 6 | 6 |
